# Effect of intracranial electrical stimulation on dynamic functional connectivity in medically refractory epilepsy

**DOI:** 10.3389/fnhum.2023.1295326

**Published:** 2023-12-20

**Authors:** Meili Lu, Zhaohua Guo, Zicheng Gao

**Affiliations:** School of Information Technology Engineering, Tianjin University of Technology and Education, Tianjin, China

**Keywords:** intracranial electrical stimulation, functional magnetic resonance imaging, dynamic functional connectivity, graph theory, epilepsy

## Abstract

**Objective:**

The objective of this study was to explore the distributed network effects of intracranial electrical stimulation in patients with medically refractory epilepsy using dynamic functional connectivity (dFC) and graph indicators.

**Methods:**

The time-varying connectivity patterns of dFC (state-based metrics) as well as topological properties of static functional connectivity (sFC) and dFC (graph indicators) were assessed before and after the intracranial electrical stimulation. The sliding window method and *k*-means clustering were used for the analysis of dFC states, which were characterized by connectivity strength, occupancy rate, dwell time, and transition. Graph indicators for sFC and dFC were obtained using group statistical tests.

**Results:**

DFCs were clustered into two connectivity configurations: a strongly connected state (state 1) and a sparsely connected state (state 2). After electrical stimulation, the dwell time and occupancy rate of state 1 decreased, while that of state 2 increased. Connectivity strengths of both state 1 and state 2 decreased. For graph indicators, the clustering coefficient, k-core, global efficiency, and local efficiency of patients showed a significant decrease, but the brain networks of patients exhibited higher modularity after electrical stimulation. Especially, for state 1, there was a significant decrease in functional connectivity strength after stimulation within and between the frontal lobe and temporary lobe, both of which are associated with the seizure onset.

**Conclusion:**

Our findings demonstrated that intracranial electrical stimulation significantly changed the time-varying connectivity patterns and graph indicators of the brain in patients with medically refractory epilepsy. Specifically, the electrical stimulation decreased functional connectivity strength in both local-level and global-level networks. This might provide a mechanism of understanding for the distributed network effects of intracranial electrical stimulation and extend the knowledge of the pathophysiological network of medically refractory epilepsy.

## 1 Introduction

Epilepsy is a neurological disorder disease characterized by paroxysms of synchronous and abnormal neurological activities of neuronal populations (Scharfman, 2007; Davis and Gaitanis, 2020; Royer et al., 2022). Patients with epilepsy may suddenly experience repeated seizures with no warning and no clear reason, and seizures typically last seconds or minutes and are usually accompanied by different clinical manifestations (Scharfman, 2007). According to the International League Against Epilepsy (ILAE), a diagnosis of epilepsy requires at least one unprovoked seizure or a risk of repeated seizures or the diagnosis of an epilepsy syndrome. In addition to the recurrence of seizures, epilepsy also causes many other adverse effects, including neurologic, cognitive, psychological, and social consequences that lower the quality of life of patients (Beghi, 2020). Epilepsy affects individuals of both sexes and all ages, and its prevalence is often slightly higher in men compared to women. It is estimated that approximately 65 million people have epilepsy worldwide. Thus, it is very important to investigate the pathophysiological properties and treatment methods of epilepsy.

Approximately 30% of patients with epilepsy have seizures persisting after attempting two or more adequately chosen medications, which is known as drug-resistant epilepsy (Kwan et al., 2009; Chen et al., 2018; Sudbrack-Oliveira et al., 2021). Surgical resection of seizure foci in the brain is the most commonly used treatment option for drug-resistant epilepsy. However, resection brings a high risk of irreversible damage to patients and is not suitable for some patients whose seizure focus cannot be safely removed (Singhal et al., 2018). Neurostimulation is an increasingly utilized therapy for drug-resistant epilepsy. By directly modulating a specific neural region, neurostimulation can regulate symptoms in a way that is reversible and adjustable, which avoids many adverse effects (Boon et al., 2009). However, despite several successful applications, the mechanisms by which brain stimulation attenuates epilepsy remain poorly understood (Thompson et al., 2020). Research has shown that the synchronization of brain activity during epileptic activity can be disrupted by high-frequency stimulation and the spread of epileptic activity to unaffected brain networks can be prevented (Piper et al., 2022). Consequently, the desynchronization of epileptic neural networks has been suggested as the potential mechanism for neural stimulation. While increasing bodies of evidence have proven that epilepsy is a disorder of brain networks, mapping the causal effects of invasive direct electrical stimulation on whole-brain measurement of the effects produced is a challenging problem. Recently, Thompson et al. (2020) established the first es-fMRI resource and presented data from human patients who underwent electrical stimulation during functional magnetic resonance imaging (es-fMRI), which offered feasible causal access to understand network-level effects of electrical stimulation.

FMRI is a common way to study brain activity by measuring changes to blood flow in the brain. A previous study has suggested that there are close relationships between epilepsy and brain functional networks disorders and between epilepsy and structural network disorders (Van Diessen et al., 2014). In most studies, brain functional networks are characterized by static functional connectivity (sFC) based on the Pearson correlation (Abraham et al., 2017). In an sFC matrix, a single measurement of connectivity is obtained across the entire time series of functional data. As a result, time-variant features will be lost in the brain functional network. To capture those dropped temporal features, the concept of dynamic functional connectivity (dFC) was proposed, which is used to characterize the inherent time-varying properties of brain networks. Research studies have shown that time-varying features of dFC are closely related to several neurological diseases such as epilepsy and Parkinson’s disease (Sahib et al., 2018; Abreu et al., 2019; Fiorenzato et al., 2019). The research of Sahib et al. verified the feasibility of the dFC method for epilepsy analysis (Sahib et al., 2018). Compared to traditional FC analysis, dFC analysis has the advantage of being able to identify additional activated brain regions in the epileptic discharge process during the inter-seizure period (Kowalczyk et al., 2020). Moreover, patients with temporal lobe epilepsy exhibit significant abnormal connectivity patterns and topological characteristics in their dFC. Using dFC methods, changes in the hippocampal network’s intrinsic temporal and functional modular patterns can be identified continuously (Li et al., 2022; Pang et al., 2022). DFC provides new insights into the pathological mechanisms of epilepsy (Klugah-Brown et al., 2019). However, the influences of electrical stimulation on dFC in epilepsy are still not clear and are seldom explored (Sawada et al., 2022; Bacon et al., 2023), which hinders the improvement of treatment effects.

**Figure 1:**
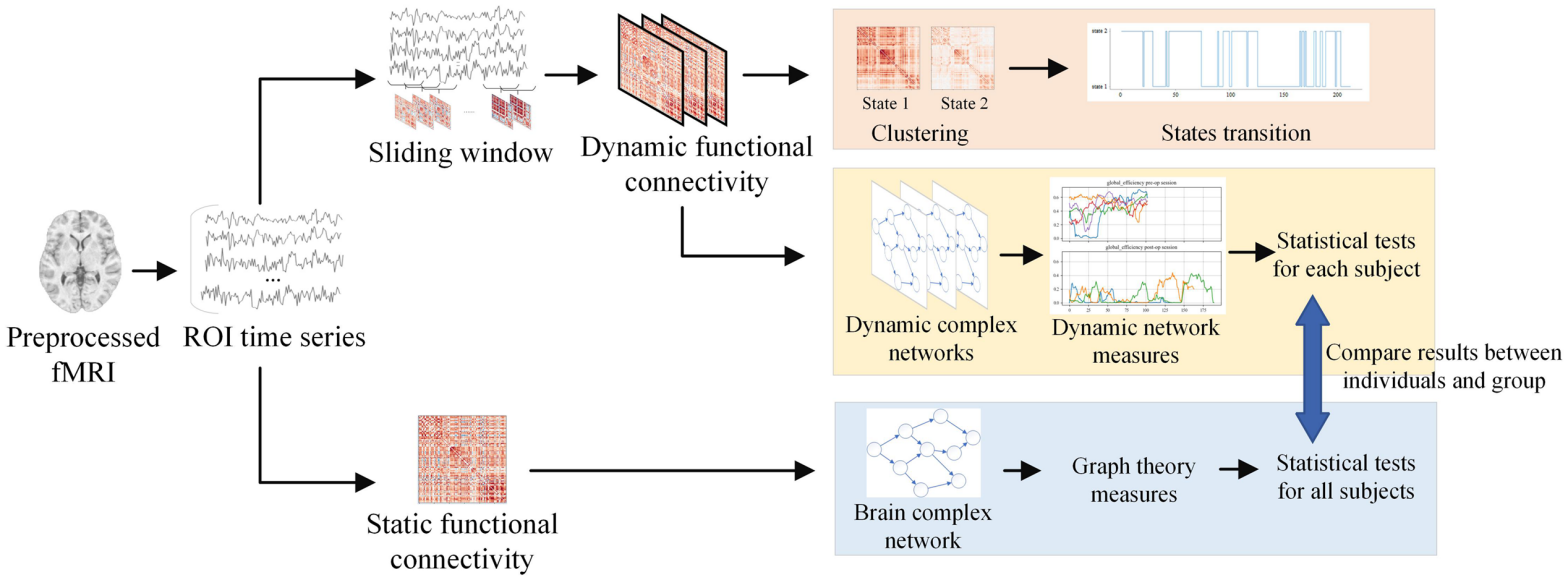
Analysis process for this study. After extracting the time series from preprocessed fMRI, the sliding window method was applied to obtain dynamic functional connectivity. By clustering the states, the brain state transitions can be computed. Dynamic complex networks were constructed using dynamic functional connectivity, and graph theory was applied to analyze the features of dynamic networks. As a comparison, static network features were extracted through static functional connectivity.

In this study, we propose to investigate the effects of intracranial electrical stimulation therapy on the epileptic brain functional networks by examining dFC properties before and after electrical stimulation. The sliding window and k-means clustering methods were employed to capture the time-varying properties of dFC. First, the parameters of the sliding window and k-means clustering methods were evaluated and adopted carefully. Second, the state-based metrics were used to describe time-varying properties of dFC before and after electrical stimulation. Third, graph theory methods were carried out to quantify the topological changes of sFC and dFC. Discussions and conclusions are given at the end of this study. The analysis process is illustrated in Figure 1.

## 2 Materials and methods

### 2.1 Dataset

In this study, we analyzed the es-fMRI dataset that was originally published by Thompson et al. (2020). The dataset resource is available in the OpenNeuro database with accession number ds002799. It is the first electrical stimulation fMRI resource for patients who were undergoing epilepsy monitoring (Thompson et al., 2020). Data were collected from 26 human patients with medically refractory epilepsy who had chosen to undergo neurosurgical treatment. Electrodes were implanted into their brains to localize the epileptic zone. Data were collected with the patient’s consent to participate and without interfering with the clinical protocol. The dataset comprises whole-brain anatomical and functional MRI scans from the eyes-open resting state before and after implantation at 3 T. Our study focused solely on analyzing fMRI data. Datasets comprising up to five sessions, with each session lasting approximately 4.8 min, before implantation (called “pre-op” session) and up to ten sessions, with each session lasting approximately 10 min, after implantation (called “post-op” session) were obtained in a subset of participants. Stimulation was performed through an isolated stimulus generator using a biphasic, charge-balanced stimulation delivered across two adjacent contacts of a depth electrode. No behavioral or experiential effects were evoked during stimulation (Thompson et al., 2020). More details about each participant and the acquisition of fMRI data can be found in Supplementary Tables S1, S2 respectively.

The data were preprocessed through a custom pipeline of fMRIPrep (RRID:SCR_016216) when it was published (Esteban et al., 2019; Thompson et al., 2020). The preprocessing steps in the custom pipeline include generating reference volume and corresponding skull-stripped version, obtaining deformation field to correct for susceptibility distortions, co-registering, regularization, head-motion estimation, slice-timing correction, resampling BOLD time series into their native space by correcting head-motion and susceptibility distortions, resampling BOLD time series into MNI152NLin2009cAsym standard space, and estimating confound time series (Esteban et al., 2019; Thompson et al., 2020). To extract time series, the AAL template for SPM12 was used to identify 116 regions of interest (ROIs) in the whole brain after preprocessing (Tzourio-Mazoyer et al., 2002). Time series were extracted using NiLearn (RRID:SCR_001362). Data were cleaned after extracting time series. Some data were removed due to being too short or lacking pre-op sessions. Only 20 participants were left after data cleaning.

### 2.2 Dynamic functional connectivity

dFC is a sequence of time-variant functional connectivity that characterizes non-stationary functional activity in brain regions. There are several methods to characterize dFC, such as the sliding window method, time-frequency analysis, and single-volume co-activation patterns (Soares et al., 2016). The sliding window method is currently the most popular method (Savva et al., 2019). In this study, we chose to use the sliding window method as well.

#### 2.2.1 Sliding window method

The sliding window method was applied to divide the time series into subsequence windows with a specific window length and step size. The functional connectivity matrix was extracted using the Pearson correlation coefficient from time series within each window. The sequences of these functional connectivity matrices are known as dFC (Hutchison et al., 2013).

The sliding window length and the step size are two parameters that may affect clustering performance (Hindriks et al., 2016). We evaluated the effects of these parameters on clustering performance to obtain the optimal parameters. Window length was varied from 30s to 180 s (step size was 1 TR, the number of clusters was 2). Step size was changed from 1 TR to 10 TR (window length was 60s, the number of clusters was 2). Python module scikit-learn (RRID: SCR_002577) was used to accomplish the parameter evaluation (Pedregosa et al., 2011).

The effects of the sliding window parameters on the quality of clustering were estimated using four different criteria. The first one is the elbow criterion. For each window length or step size, the inertia values of K-means clustering were plotted. Inertia value is the measure of the internal coherence properties of each cluster here (Pedregosa et al., 2011; Bijsterbosch, 2017). Then, the parameters corresponding to the elbow point of the inertia values were chosen as the optimal parameters. Because this method is an empirical conclusion, other evaluation indicators are used in conjunction with it to make comprehensive decisions. The second method is the silhouette coefficient. The silhouette coefficients of each sample were calculated using clustering results, and their mean value was computed as the overall silhouette coefficient. The formula for the silhouette coefficient of a single sample is written as follows (Rousseeuw, 1987):

$$s\left(i\right)=\frac{b\left(i\right)-a\left(i\right)}{\max\left\{a\left(i\right),b\left(i\right)\right\}},\;\left(1\right)$$

where $s\left(i\right)$ denotes the silhouette coefficient of sample i , $a\left(i\right)$ denotes the average Euclidean distance of sample i to all the other objects of the cluster to which it has been assigned, and $b\left(i\right)$ denotes the minimum average dissimilarity of sample i to all the other clusters (Rousseeuw, 1987). The dissimilarity was defined as the Euclidian distance in our study. Clustering performs better when $s\left(i\right)$ approaches 1. The third method is the Calinski–Harabasz index. The score of the Calinski–Harabasz index is defined as the ratio of the sum of between-cluster dispersion and the sum of within-cluster dispersion for all clusters (Calinski and Harabasz, 1974). The Calinski–Harabasz index for n samples and K clusters is as follows (Maulik and Bandyopadhyay, 2002):

$$CH=\left[\frac{\sum_{k=1}^{K}n_{k}∥z_{k}-z{∥}^{2}}{K-1}\right]/\left[\frac{\sum_{k=1}^{K}\sum_{i=1}^{n_{k}}∥x_{i}-z_{k}{∥}^{2}}{n-K}\right],\;\left(2\right)$$

where $n_{k}$ is the number of samples in cluster k , $z_{k}$ is the centroid of cluster k , and z is the centroid of all data (Maulik and Bandyopadhyay, 2002). A higher Calinski–Harabasz score results in better clustering (Calinski and Harabasz, 1974). The fourth method is the Davies–Bouldin index. It represents the average similarity between clusters and is defined as the ratio of the sum of within-cluster scatter to between-cluster separation (Davies and Bouldin, 1979; Maulik and Bandyopadhyay, 2002). The Davies–Bouldin index is computed as follows (Davies and Bouldin, 1979):

$$DB=\frac{1}{k}\overset{k}{\underset{i=1,i\neq j}{\sum }}\max\left(R_{ij}\right)\;\left(3\right)$$

$$R_{ij}=\frac{s_{i}+s_{j}}{d_{ij}},\;\left(4\right)$$

where k denotes the number of clusters, $s_{i}$ denotes the cluster diameter of cluster i , which is the average distance between each sample and cluster centroid, and $d_{ij}$ denotes the distance between centroids of cluster i and cluster j . A better partition should have a lower Davies–Bouldin index value (Davies and Bouldin, 1979).

### 2.3 Clustering and states analysis

After selecting a set of sliding window parameters and extracting dFC from the ROI time series, the impact of the target number of states on clustering quality was evaluated. Different states were created by clustering functional connectivity matrices in dFC using the K-means algorithm. Considering that the randomness in the initial cluster centroid selection may affect the quality of clustering, K-means was iterated 10 times with randomly generated initial cluster centroids to reduce the bias caused by initial random selection of cluster centroids. Then, we evaluated the effects of the number of FC states on the quality of clustering by varying k-score from 2 to 10 (window length 60s, 1TR) using the four criteria mentioned above to determine the optimal number of FC states.

The dFC windows of all participants were clustered using K-means. Inspired by the studies of Damaraju et al. (2014) and Fiorenzato et al. (2019), the centroids of each state of dFCs were plotted for pre-op and post-op sessions. Then, three indexes were calculated for state-based analysis of dFC, namely, dFC strength, dwell time, and occupancy rate. Here, dFC strength is defined as the average of the functional connectivity values between ROIs; dwell time is defined as the average duration time of each participant in each state; and the occupancy rate is defined as the percentage of time that each participant is present in each state (Iraji et al., 2021).

After index calculation, statistical analysis methods were applied to dwell time and occupancy rate to evaluate the statistically significant change after electrical stimulation. Generally, the student t-test is commonly used in statistical analysis. However, sometimes, experimental data do not meet the preconditions of *t*-test such as normal distribution and homogeneity of variance. To determine whether the preconditions were met, the Shapiro–Wilk test can be used for normality testing, and the Levene test for equal variances can be used for the homogeneity of variance testing. We computed the Shapiro–Wilk test and Levene test and found that the abovementioned preconditions in the es-fMRI data were not suited for the t-test. As a result, the Mann–Whitney U-test was adopted for this study. It is a non-parametric testing method that does not require any prerequisite assumptions. In the study, we assumed that there was no significant change after stimulation. This hypothesis will be rejected if the *p*-value of the test is less than 0.05. A rank comparison would be tested if there were significant differences between pre-op and post-op sessions. Python modules SciPy (RRID: SCR_008058) and statsmodels (RRID: SCR_016074) were used for statistical analysis (Seabold and Perktold, 2010; Virtanen et al., 2020).

### 2.4 Graph theory and statistical analysis

A complex network is commonly used to describe the connections between brain regions. Graph theory is a mathematical method that is commonly used to study complex networks (Shen et al., 2010). Graph theory provides a framework for simulating pairwise communication between network elements. A binary functional connectivity matrix was used to construct brain complex networks in this study. The functional connectivity matrix was binarized into a 0–1 matrix by setting an appropriate threshold. The binary functional connectivity matrix could be considered as an adjacency matrix of the brain network. After building complex brain networks, several graph indicators (clustering coefficient, *k*-core, modularity, global efficiency, local efficiency, and network assortativity) were calculated (Rubinov and Sporns, 2010). Non-parametric statistical analysis (based on the Mann–Whitney U-test) was applied to these measures to test for significant differences after electrical stimulation. All of these graph indicators were drawn as violin plots. In this study, the clustering coefficient represents the average of all the nodes’ clustering coefficients, while k-core represents the maximum core count of the k-core subgraph.

Inspired by the idea of dFC, we propose a dynamic graph theory method to explain the time-varying features of brain networks. First, a series of graph indicators for complex brain networks were calculated based on dFC. Second, a multi-layer network was constructed based on binary dFC. Each layer of the network corresponds to the binary functional connectivity matrix of a window in dFC. Third, a series of graph indicators were calculated and plotted on a line chart. Lastly, non-parametric statistical tests were utilized to examine which graph metric experienced a significant change after each participant underwent electrical stimulation. The stationarity of dynamic graph indicators was further tested using the Augmented Dickey–Fuller (ADF) unit root test and the Kwiatkowski–Phillips–Schmidt–Shin (KPSS) test. Periodic analysis of dynamic graph indicators was also performed using Fast Fourier Transform (FFT) as well. Calculations were done using NetworkX (RRID: SCR_016864) (Hagberg et al., 2008). Dynamic measures were plotted using MatPlotLib (RRID: SCR_008624) (Caswell et al., 2022).

## 3 Results

### 3.1 Sliding window parameters

**Figure 2:**
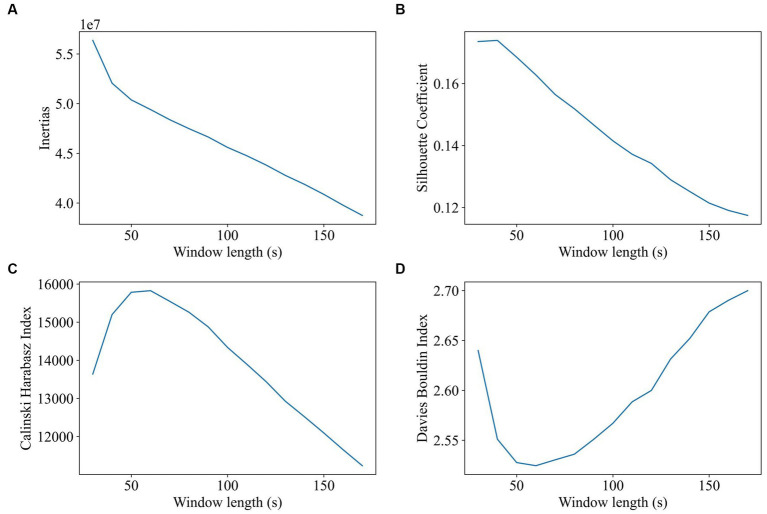
Impact of window length on clustering (window length ranges from 30 to 180, 1TR a step, 2 states). The goal of subplot **(A)** is to locate the elbow point of the inertias. The goal of subplot **(B)** is to obtain the maximum value of the silhouette coefficient. The goal of subplot **(C)** is to find the highest value of the Calinski–Harabasz index. The goal of subplot **(D)** is to find the minimum value of the Davies–Bouldin index.

Window length and step size of the sliding window method were estimated using the elbow criterion, silhouette coefficient, Calinski–Harabasz index, and Davies–Bouldin index. Window length was tested and reported to be varying from 30s to 180 s (step size was 1 TR, and the number of clusters was 2). The evaluation results of the impact of window length on clustering are shown in Figure 2. All the participants’ dFC were used for clustering. The results of the Calinski–Harabasz and Davies–Bouldin indexes show that the best option is between 40s and 80s.

**Figure 3:**
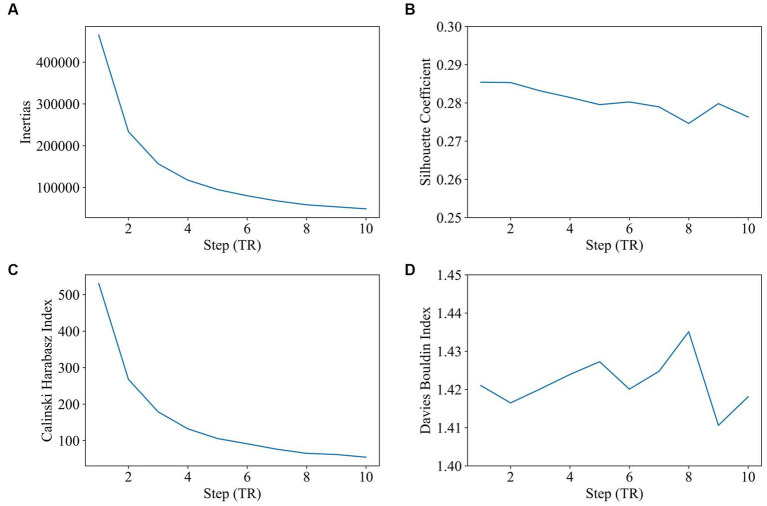
Impact of step size on clustering (step size range: 2–10, window length: 60 s, 2 states). The goal of subplot **(A)** is to locate the elbow point of the inertias. The goal of subplot **(B)** is to obtain the maximum value of the silhouette coefficient. The goal of subplot **(C)** is to find the highest value of the Calinski–Harabasz index. The goal of subplot **(D)** is to find the minimum value of the Davies–Bouldin index.

Step size was tested varying and reported to be from 1 TR (repetition time) to 10 TR (window length was 60s, and the number of clusters was 2). As shown in Figure 3, step size has very little impact on clustering. There is a slight advantage to choosing a shorter step size, since a shorter step size makes the dFC series contain more time-variant information. The step size of 1 TR will be selected in the future study.

### 3.2 Analysis of the states

**Figure 4:**
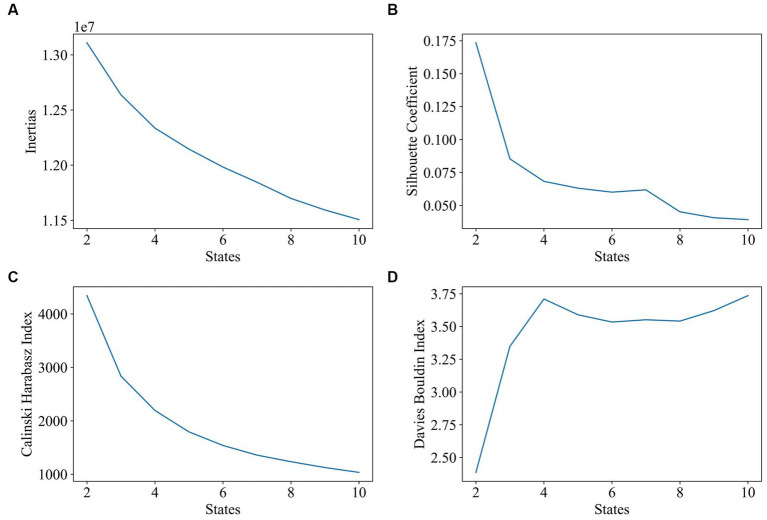
Impact of state number on clustering (number of states range: 2–10, window length: 60 s, 1TR). The goal of subplot **(A)** is to locate the elbow point of the inertias. The goal of subplot **(B)** is to obtain the maximum value of the silhouette coefficient. The goal of subplot **(C)** is to find the highest value of the Calinski–Harabasz index. The goal of subplot **(D)** is to find the minimum value of the Davies–Bouldin index.

The first step was to determine the impact of the target number of states on clustering. Figure 4 displays the evaluation results of clustering with states that range from 2 to 10. Based on the results of the silhouette coefficient, Calinski–Harabasz index, and Davies–Bouldin index, the optimal number of target states is 2.

**Figure 5:**
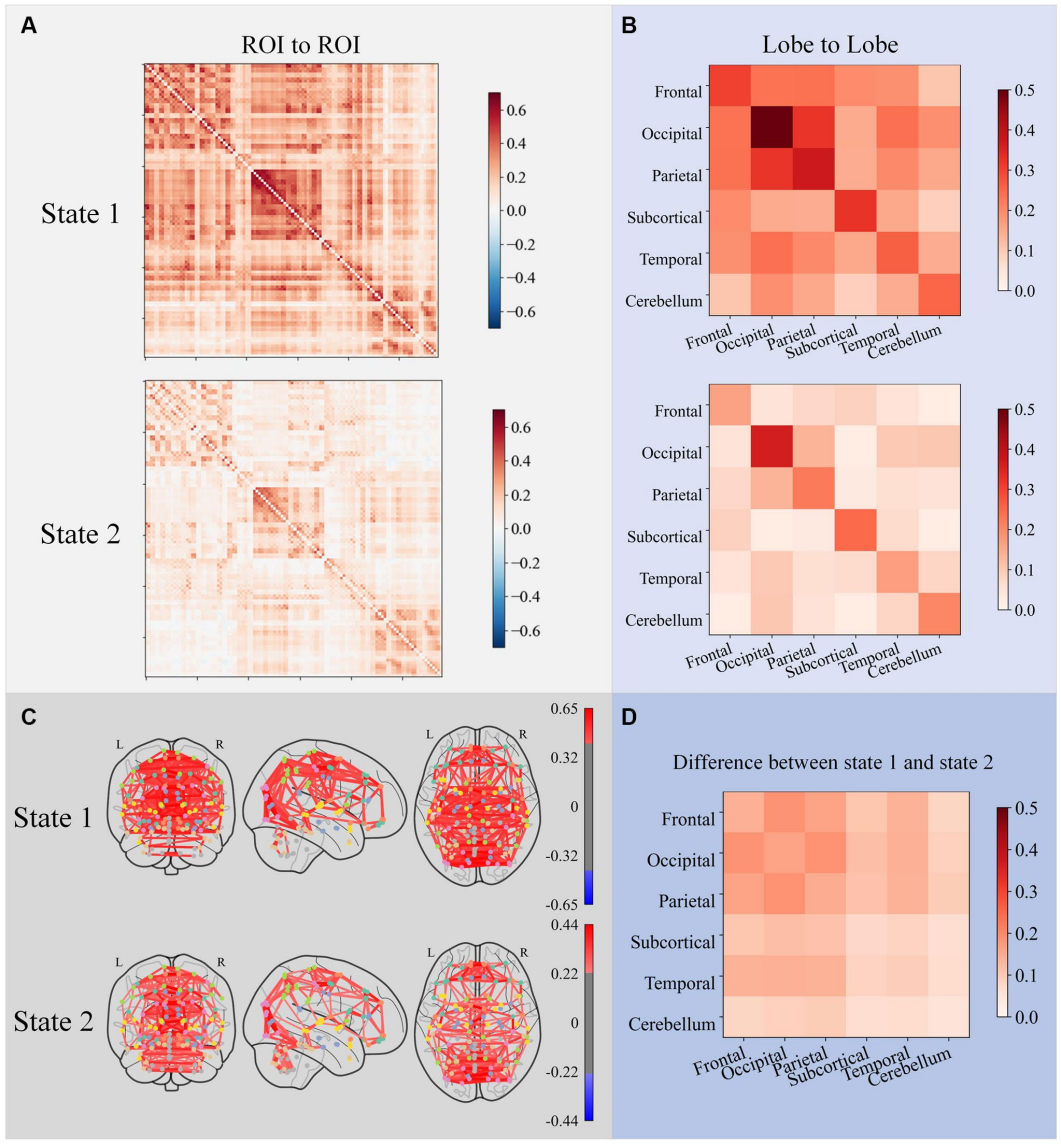
ROI-to-ROI FC matrices of cluster centers for each state subplot **(A)**, lobe-to-lobe FC matrices of cluster centers for each state subplot **(B)**, and brain network connectome corresponding to each FC matrix subplot **(C)**. Only connections with the top 5% strength were plotted in connectomes. The difference between the two states subplot **(D)** shows that all lobe-to-lobe connections in state 1 have greater strength than in state 2.

Two states were formed using the K-means algorithm for clustering all participants’ dFCs. The subplot A in Figure 5 shows the cluster centroids. The 116 ROIs were divided into six brain lobes based on their anatomical positions: the frontal, occipital, parietal, subcortical, temporal, and cerebellum lobes. Subplot B in Figure 5 displays the average functional connectivity (the average value of the Pearson correlation coefficient) between all six lobes. Subplot C in Figure 5 shows the brain network connectome that corresponds to each state. Subplot D in Figure 5 shows that all lobe-to-lobe connections in state 1 have greater strength than in state 2. The dFC strength of state 1 was 0.214 and state 2 was 0.079.

**Figure 6:**
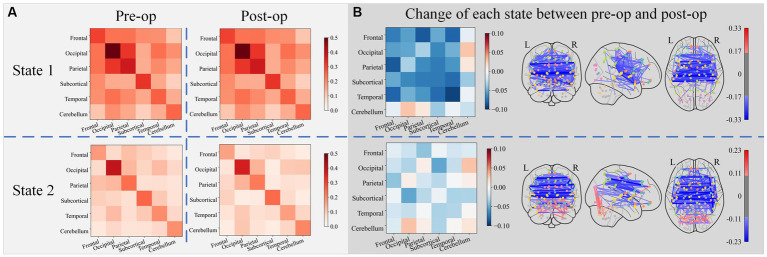
The lobe-to-lobe centroid FC matrix group by state for the pre-op and post-op session subplot **(A)**, along with their changes (obtained by subtracting pre-op FC matrices from post-op FC matrices) subplot **(B)**. Only connections with the top 5% strength change were plotted in connectomes (blue edges denote the decrease of connectivity and red edges denote the increase of connectivity).

Figure 6 shows the lobe-to-lobe centroid functional connectivity matrix grouped by state for pre-op and post-op sessions as well as their changes. Electrical stimulation resulted in a significant decrease in connectivity within the temporal lobe, connectivity between the frontal lobe and parietal lobe, and connectivity between the frontal lobe and temporal lobe in state 1. Electrical stimulation resulted in an increase in the connectivity between the occipital and cerebellum lobes. In state 2, the connectivity between the occipital lobe and the subcortical lobe decreased the most, and the connectivity between the occipital lobe and the cerebellum lobe increased the most after electrical stimulation.

**Figure 7:**
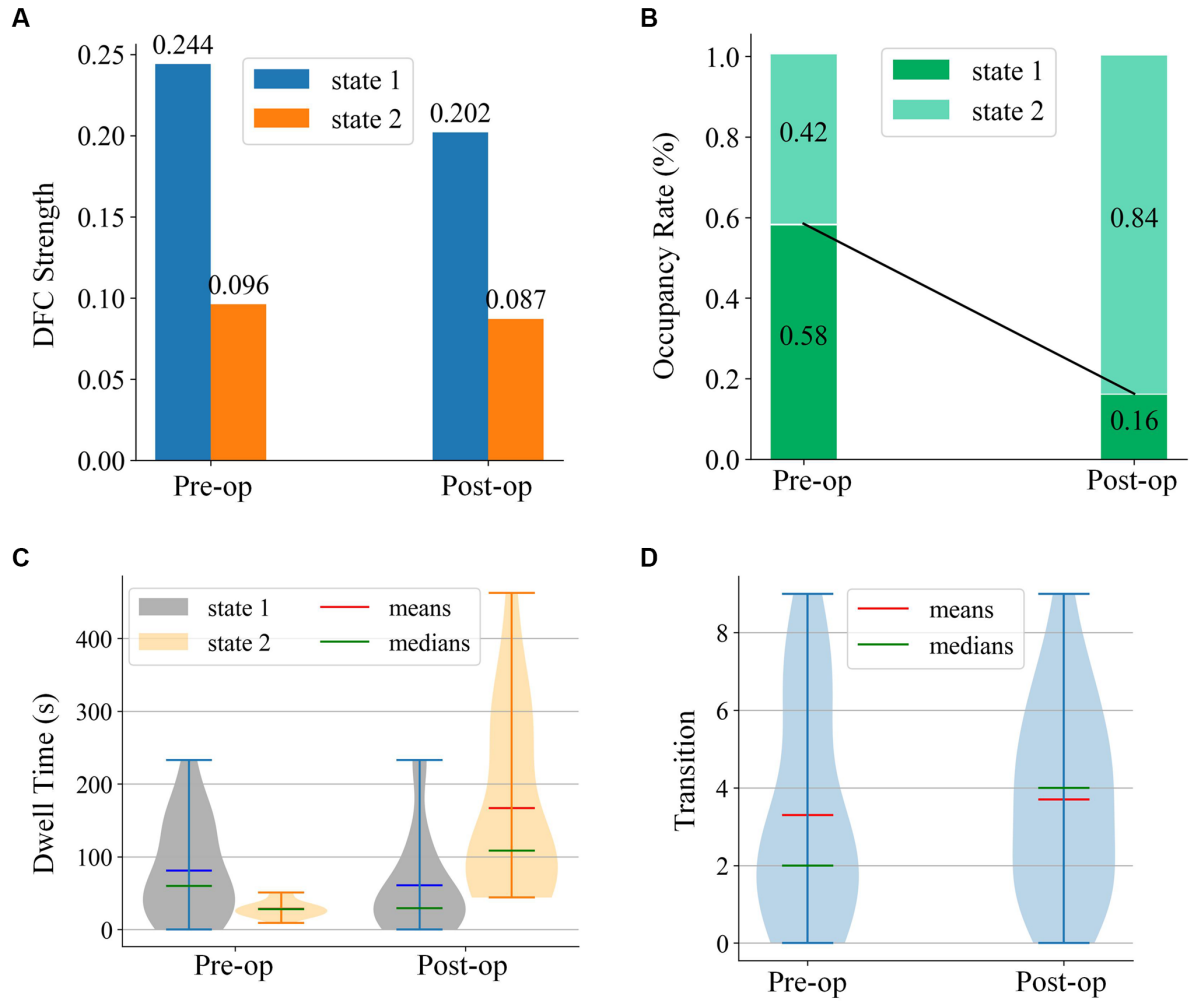
Four states analysis metrics for state centroids of pre-op and post-op sessions.

Figure 7 illustrates the dFC strength, occupancy rate, dell time, and transitions of the two states before and after electrical stimulation. Figure 7 clearly indicates that the dFC strength for both states decreased in the post-op session. The occupancy rate of state 1 decreased from 0.58 to 0.16. Relatively, the occupancy rate of state 2 increased from 0.42 to 0.84. The Mann–Whitney U-tests and rank comparison results showed that the occupancy rate of state 1 had decreased (mean values of the pre-op session and post-op session are 0.57 and 0.16, statistic = 3.25, *p* = 0.004 < 0.05) and state 2 increased (mean values of the pre-op session and post-op session are 0.43 and 0.84, statistic = −3.25, *p* = 0.004 < 0.05). As can be seen in Figure 7, there is very little difference between the dwell time of the two states in the pre-op session. The dwell time of state 2 has grown significantly longer than that of state 1 in the post-op session. The statistical tests and rank comparison results showed that the dwell time of state 1 decreased (mean values of the pre-op session and post-op session are 77.17 s and 28.48 s, statistic = 2.27, *p* = 0.03 < 0.05) and dwell time of state 2 increased (mean values of the pre-op session and post-op session were 69.63 s and 353.10s, statistic = −4.02, *p* < 0.001). The violin plot of transition in Figure 7 shows that the transitions per minute did not significantly change after electrical stimulation. Only the medians of transition changed from 2 to 4.

### 3.3 Graph indicators

Figure 7 displays the violin plots of the sFC graph indicators for all participants. The statistical test results for these measures are listed in Table 1. It can be seen from Figure 7 that the k-core, clustering, global efficiency, and local efficiency in the post-op session have all decreased compared to that of the pre-op session, while modularity has increased. There was no significant change in the network assortativity. This conclusion is consistent with the statistical test results.

**Table 1:** The Mann–Whitney *U*-test for graph indicators of sFC.

	Mean value		Statistics	*p* value
	Pre-op	Post-op		
K-core	18.27	10.78	3.578	0.001
Clustering coefficient	0.50	0.43	3.354	0.001
Modularity	0.40	0.54	−4.357	< 0.001
Network assortativity	0.36	0.37	−0.441	0.570
Global efficiency	0.36	0.24	5.038	< 0.001
Local efficiency	0.61	0.52	3.866	< 0.001

Each participant’s dynamic graph indicators were tested individually using statistical tests. Table 2 summarizes the number of participants with statistical changes in the graph indicators. In total, 16 out of the 20 participants showed a decrease in clustering coefficient, k-core, global efficiency, and local efficiency, while the other four participants showed an increase in those metrics. In total, 12 out of the 20 participants experienced a slight decrease in their network assortativity, while four participants experienced an increase and four participants experienced no change. In total, 13 out of the 20 participants decreased in modularity, while the other seven participants increased their modularity.

**Table 2:** Number of participants with statistical changes in graph indicators.

	No statistical changes	Increased	Decreased
K-core	0	4	16
Clustering coefficient	0	4	16
Modularity	0	13	7
Network assortativity	4	4	12
Global efficiency	0	4	16
Local efficiency	0	4	16

After conducting stationarity analysis on dynamic graph indicators, we found that the graph indicators of some participants altered steadily over time, while others were unstable. The graph indicators of most participants were consistent across multiple acquisition runs. Most participants had consistent stationarity before and after stimulation, with only a few participants exhibiting differences. Moreover, there were no significant periodic trends observed after conducting a periodic analysis.

## 4 Discussion

In this study, we evaluated the change in dynamic functional connectivity before and after electrical stimulation as well as the choice of parameters for gaining dFC. The best clustering quality is achieved by selecting a window length of approximately 60s, which is consistent with prior findings that a window length of 30s–60s can successfully capture dFC variations (Preti et al., 2017). According to our study, two distinct states were discriminated based on k-means clustering and the performance evaluation of state numbers on clustering. These results are similar to Li et al.’s study on generalized tonic clone sizes that dFC was also clustered into two states: state 1 with strong positive international interactions and state 2 with weak functional connectivity (Y. Li et al., 2023). In the pre-op session, state 1 has a stronger functional connectivity and a higher occupancy rate than that of state 2, but there is not much difference in dwell time between the two states. In the post-op session, electrical stimulation brings about a decline in the dwell time and occupancy rate of state 1 but an increase in that of state 2. Specifically, state 1’s occupancy rate decreased by 42%, and the decline in the dwell time of state 1 as well as the increase in the dwell time of state 2 make the dwell time of state 2 last longer than that of state 1. The number of transitions per minute for each state did not differ significantly before and after electrical stimulation, indicating that electrical stimulation has a minor effect on the frequency of state changes. Other research studies have found that, following initial intracranial brain stimulation, there is a decrease in brain network switching and synchronization, but this gradually stabilizes over time (Pedersen and Zalesky, 2021).

Our study indicates that functional connections in both centroids of state 1 and state 2 in the pre-op session are stronger than those in the post-op session. Previous research suggests that the seizure onset zone of most participants may be in the frontal lobe and temporal lobe (Thompson et al., 2020), and several studies have reported a significant increase in functional connectivity within the medial temporal lobe, within the frontal lobe, and between the parietal and frontal lobes in temporal lobe epilepsy patients when compared to healthy controls (Liao et al., 2010; Haneef et al., 2014). To investigate the functional connectivity changes, we divided the 116 ROIs into six lobes according to their anatomical location (Ganella et al., 2017). We found that there were decreases in functional connectivity within the temporal lobe (approximately 0.09, estimated by Pearson’s correlation coefficient), between the frontal lobe and parietal lobe (approximately 0.08), and between the frontal lobe and temporal lobe (approximately 0.08) of state 1. It is inferred that the effects of the electrical stimulation on suppressing epileptic activities in the brain of patients may manifest in decreasing the functional connectivity of the seizure onset zone.

The changes in graph indicators for sFC and dFC also indicate that electrical stimulation may result in a statistical decrease in brain functional connectivity. Other studies have shown that epilepsy patients have an increase in local network measures (local efficiency and cluster coefficient) and a decrease in global network measures (global efficiency) compared to healthy controls (Pedersen et al., 2015). Our study found that stimulation causes a decrease in these measures. This may serve as evidence for the hypothesis that electrical stimulation can treat epilepsy by disrupting the epileptic network (Chari et al., 2020; Piper et al., 2022). Although stimulation reduces abnormally high local connectivity, it cannot recover abnormally low global connectivity. With the proposed dynamic graph theory, it is feasible to conduct statistical tests on individual participants and apply time series analysis to graph indicators. However, there are four participants whose statistical analysis results were different from the others. This might be caused by individual differences or the poor effectiveness of the electrical stimulation scheme. We are currently unable to make a conclusion on this. Thus, further analysis of the research on the electrical stimulation scheme and its curative effects is required.

Our study found that no common characteristic was identified even after conducting several time series analyses on dynamic graph indicators for each participant. Periodic or seasonal patterns were not observed for most participants. Furthermore, no statistical principles governing stationarity and trend changes have been discovered. There may be no rule for short-term periodic alteration in the resting state brain functional network of epilepsy. However, considering that epilepsy seizures occur periodically, it is uncertain whether there is a periodic pattern in the brain network over the long term.

## 5 Conclusion

In this study, we investigated the distributed network effects of cerebral electrical stimulation on patients with medically resistant epilepsy using time-varying connectivity patterns of dynamic functional connectivity (dFC) and graph indicators. Two states of connectivity were identified. State 1 has a stronger connectivity pattern, while state 2 has a weaker connectivity pattern. After electrical stimulation, state 1 had a decline in dwell time and occupancy rate, but state 2 had an increase. There was no significant difference in the frequency of transition for both states between the pre-op session and post-op session. Intracranial electrical stimulation tends to put the brain network in a state with lower strength and weakens the functional connectivity strength of both states. On the whole, intracranial electrical stimulation can significantly reduce the functional connectivity of the brain network in both the whole brain and local areas, especially the functional connectivity within and between the frontal and temporary lobes.

Our study assessed the topological patterns of the brain network using graph theory indicators and statistical tests. The results show that the clustering coefficient, k-core, global efficiency, and local efficiency decreased significantly. Dynamic graph indicators make it possible to apply statistical tests and time series analysis to each participant individually. There are several patients whose individual statistical analysis is different from group analysis. This indicates that electrical stimulation therapy may not have the same effect on everyone, and there may be individual differences. This supports the idea that personalized electrical stimulation incorporating dynamic functional information derived from participant data is necessary for clinical treatment.

## Data availability statement

The original contributions presented in the study are included in the article/Supplementary material, further inquiries can be directed to the corresponding author/s.

## Ethics statement

Ethical approval was not required for the study involving humans in accordance with the local legislation and institutional requirements. Written informed consent to participate in this study was not required from the participants or the participants’ legal guardians/next of kin in accordance with the national legislation and the institutional requirements.

## Author contributions

ML: Conceptualization, Funding acquisition, Methodology, Supervision, Writing – review & editing. ZhG: Methodology, Visualization, Writing – original draft, Writing – review & editing. ZiG: Validation, Visualization, Writing – review & editing.
