# Supplementary material for: Effect of intracranial electrical stimulation on dynamic functional connectivity in medically refractory epilepsy
Source: Front Hum Neurosci. 2023 Dec 20;17:1295326. doi: 10.3389/fnhum.2023.1295326 (PMC10765510; doi:10.3389/fnhum.2023.1295326)
Supplement: Supplementary file 1 [file Data_Sheet_1.docx]

Supplementary Material

# Supplementary Figures


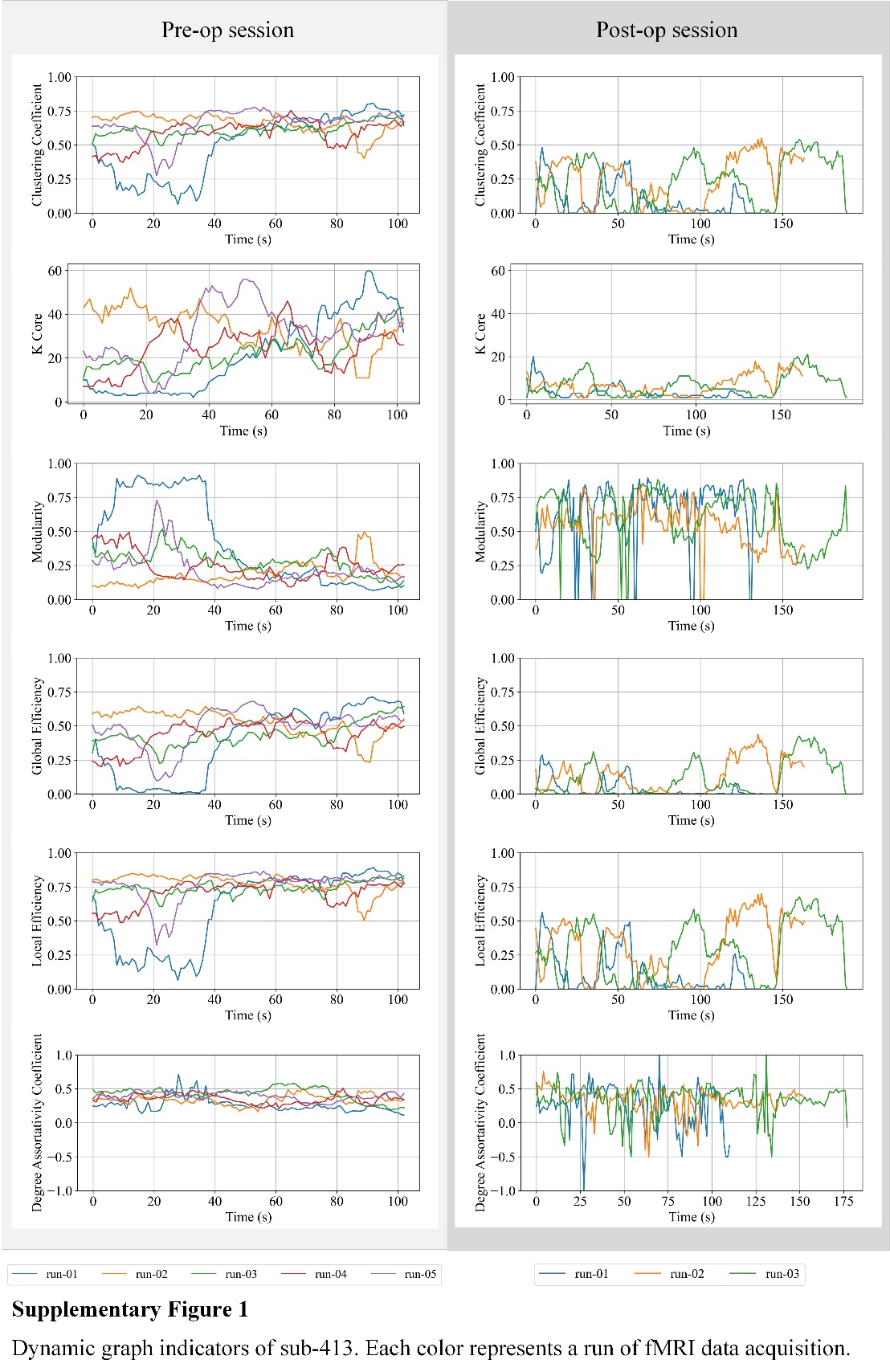


# Supplementary Tables

Supplementary Table 1 Information about each subject.

| Participant id | Sex | Age | Handedness | Seizure onset zone |
| --- | --- | --- | --- | --- |
| 292 | Female | 50 | Left | Left mesial temporal lobe, Left frontal lobe |
| 294 | Male | 34 | Right | Right anterior frontal lobe |
| 302 | Female | 47 | Right | Left mesial temporal lobe |
| 303 | Female | 34 | Right | Right mesialtemporal lobe, Left mesial temporal encephalomalacia |
| 307 | Male | 30 | Right | Left insula |
| 314 | Female | 30 | Right | Bilateral mesial temporal lobe |
| 316 | Female | 31 | Right | Right mesial temporal lobe |
| 320 | Female | 50 | Right | Right hippocampus |
| 330 | Male | 43 | Left | Left occipital lobe |
| 331 | Male | 35 | Right | Left mesial temporal lobe |
| 334 | Male | 39 | Left | Right temporal pole, Left temporal base |
| 335 | Male | 31 | Right | Bilateral mesial temporal lobe |
| 352 | Male | 31 | Right+Left | Left frontal cystic mass |
| 372 | Male | 34 | Right | Left temporal pole |
| 376 | Female | 48 | Right | Right mesial temporal lobe |
| 384 | Male | 38 | Right | Right mesial temporal lobe, Right frontal pole |
| 399 | Female | 22 | Right | Right mesial temporal lobe, Possible right frontal base |
| 400 | Male | 59 | Right | Left mesial temporal lobe |
| 405 | Male | 19 | Right | Left frontal encephalomalacia |
| 413 | Male | 22 | Left | Right mesial temporal lobe |

Supplementary Table 2 Information about fMRI data acquisition.

| **Subjects** | **Session** | **TR (s)** | **Number of Runs** | **Stimulation Electrode Group** | **Stimulated Hemisphere** |
| --- | --- | --- | --- | --- | --- |
| **292** | Preop. (resting state) | 2 |  |  |  |
|  | Postop. (es-fMRI) | 3.1 | 3 | Posterior medial frontal depth | Left |
|  |  |  | 2 | Heschls gyrus depth | Left |
| **294** | Preop (resting state) | 2 |  |  |  |
|  | Postop. (es-fMRI) | 3 | 2 | Amygdala depth | Right |
| **302** | Preop (resting state) | 2 |  |  |  |
|  | Postop. (es-fMRI) | 3 | 1 | Heschls gyrus depth | Left |
|  |  |  | 1 | Amygdala depth | Left |
| **303** | Preop (resting state) | 2.26 |  |  |  |
|  | Postop. (es-fMRI) | 3 | 5 | Amygdala depth | Right |
| **307** | Preop (resting state) | 2.26 |  |  |  |
|  | Postop. (es-fMRI) | 3 | 5 | Heschls gyrus depth | Left |
|  |  |  | 2 | Amygdala depth | Left |
| **314** | Preop (resting state) | 2.26 |  |  |  |
|  | Postop. (es-fMRI) | 3 | 1 | Middle cingulate depth | Left |
|  |  |  | 1 | Heschls gyrus depth | Left |
|  |  |  | 1 | Temporal grid | Left |
|  |  |  | 1 | Amygdala depth | Left |
|  |  |  | 1 | Anterior cingulate depth | Left |
| **316** | Preop (resting state) | 2.26 |  |  |  |
|  | Postop. (es-fMRI) | 3 | 3 | Heschls gyrus depth | Right |
|  |  |  | 3 | Amygdala depth | Right |
|  |  |  | 1 | Posterior hippocampal depth | Right |
| **320** | Preop (resting state) | 2.26 |  |  |  |
|  | Postop. (es-fMRI) | 3 | 2 | Frontal grid | Right |
|  |  |  | 2 | Heschls gyrus depth | Right |
|  |  |  | 2 | Amygdala depth | Left |
| **330** | Preop (resting state) | 2.26 |  |  |  |
|  | Postop. (es-fMRI) | 3 | 1 | Parietal grid | Right |
|  |  |  | 2 | Amygdala depth | Right |
|  |  |  | 2 | Planum temporal depth | Right |
|  |  |  | 1 | Amygdala depth & Planum temporal depth simultaneously | Right |
|  |  |  | 1 | Superior posterior occipital depth | Right |
| **331** | Preop (resting state) | 2.26 |  |  |  |
|  | Postop. (es-fMRI) | 3 | 4 | Heschls gyrus depth | Right |
|  |  |  | 1 | Planum temporale depth | Right |
|  |  |  | 1 | Posterior hippocampal depth | Right |
|  |  |  | 1 | Frontal grid | Right |
|  |  |  | 1 | Amygdala depth | Left |
|  |  |  | 1 | Amygdala depth | Right |
|  |  |  | 1 | Amygdala depth | Left and Right |
| **334** | Preop (resting state) | 2.26 |  |  |  |
|  | Postop. (es-fMRI) | 3 | 4 | Planum temporale depth | Right |
|  |  |  | 3 | Amygdala depth | Left |
|  |  |  | 1 | Amygdala depth | Right |
|  |  |  | 1 | Posterior hippocampal depth | Right |
|  |  |  | 1 | Amygdala depth | Left and Right |
| **335** | Preop (resting state) | 2.26 |  |  |  |
|  | Postop. (es-fMRI) | 3 | 3 | Heschls gyrus depth | Right |
|  |  |  | 2 | Amygdala depth | Left |
|  |  |  | 2 | Anterior insula depth | Left |
|  |  |  | 1 | Posterior insula depth | Left |
|  |  |  | 1 | Parahippocampal strip | Left |
|  |  |  | 1 | Temporal grid | Right |
| **352** | Preop (resting state) | 2.26 |  |  |  |
|  | Postop. (es-fMRI) | 3 | 7 | Amygdala depth | Left |
|  |  |  | 2 | Heschls gyrus depth | Left |
| **372** | Preop (resting state) | 2.26 |  |  |  |
|  | Postop. (es-fMRI) | 3 | 4 | Heschls gyrus depth | Left |
|  |  |  | 3 | Amygdala depth | Left |
| **376** | Preop (resting state) | 2.26 |  |  |  |
|  | Postop. (es-fMRI) | 3.06 | 1 | Amygdala depth | Right |
|  |  |  | 2 | Frontal grid | Right |
|  |  |  | 1 | Posterior insula depth | Right |
|  |  |  | 1 | Heschls gyrus depth | Right |
| **384** | Preop (resting state) | 2.26 |  |  |  |
|  | Postop. (es-fMRI) | 3 | 4 | Amygdala depth | Right |
| **399** | Preop (resting state) | 2.26 |  |  |  |
|  | Postop. (es-fMRI) | 3 | 4 | Anterior cingulate genu depth | Right |
|  |  |  | 2 | Amygdala depth | Right |
|  |  |  | 2 | Heschls gyrus depth | Right |
| **400** | Preop (resting state) | 2.26 |  |  |  |
|  | Postop. (es-fMRI) | 3 | 2 | Heschls gyrus depth | Left |
|  |  |  | 1 | Amygdala depth | Left |
|  |  |  | 1 | Parietal - middle cingulate depth | Left |
|  |  |  | 1 | Posterior hippocampus depth | Left |
| **405** | Preop (resting state) | 2.26 |  |  |  |
|  | Postop. (es-fMRI) | 3 | 2 | Posterior insula depth | Left |
|  |  |  | 1 | Amygdala depth | Right |
|  |  |  | 1 | Amygdala depth | Left |
|  |  |  | 1 | Hippocampus depth | Right |
|  |  |  | 1 | Anterior insula orbitofrontal cortex depth | Left |
| **413** | Preop (resting state) | 2.26 |  |  |  |
|  | Postop. (es-fMRI) | 3 | 1 | Frontal operculum ofc depth | Right |
|  |  |  | 1 | Cingulate genu depth | Right |
|  |  |  | 1 | Inferior posterior insula depth | Right |
